# Eclampsia-Associated Posterior Reversible Encephalopathy Syndrome (PRES) Complicated by Intracerebral Hemorrhage: A Case Report and Review of Management Strategies

**DOI:** 10.7759/cureus.41147

**Published:** 2023-06-29

**Authors:** Hina Rashid, Muhammad Salman Saleem, Somasekhar Podile, Mohammad Saad Javaid, Shaniah S Holder, Muhammad Umer Shafique

**Affiliations:** 1 Faculty of Medicine, Dow International Medical College, Dow University of Health Sciences, Karachi, PAK; 2 House Officer, Lahore General Hospital, Lahore, PAK; 3 Internal Medicine, Nimra Institute of Medical Sciences, Vijayawada, IND; 4 Internal Medicine, Jackson Park Hospital and Medical Center, Chicago, USA; 5 General Physician, Lahore General Hospital/Ameer ud din Medical College, Lahore, PAK; 6 Medicine, American University of Barbados School of Medicine, Bridgetown, BRB; 7 Medicine, Lahore General Hospital, Lahore, PAK

**Keywords:** posterior reversible encephalopathy syndrome, ct and mri brain, generalized tonic-clonic seizures, intracerebral hemorrhage, preeclampsia-eclampsia

## Abstract

Posterior reversible encephalopathy syndrome (PRES) is a clinicoradiological syndrome that is being increasingly recognized due to the advancements in brain imaging, specifically MRI. This syndrome is characterized by symptoms including, but not limited to, headache, seizures, altered mental status, and vision loss. There are various underlying etiologies which lead to PRES occurrence; the etiology of focus in this report is preeclampsia and eclampsia. PRES is associated with the development of various types of intracerebral hemorrhage which can lead to detrimental and even fatal consequences in a patient. In our case, a 22-year-old female developed PRES within one week postpartum, which was complicated by parenchymal hemorrhage development in the fronto-parietal lobe. MRI findings were characteristic for PRES with multiple subcortical hyperintensities within the bilateral occipital lobe. The patient improved symptomatically after management with intravenous fluids, antihypertensives, antiepileptics, and antibiotics. This report aims to explore the association between preeclampsia/eclampsia (PE/E) and PRES and underscore the importance of prompt diagnosis and treatment, which can lead to recovery within a week and significantly reduce morbidity and mortality.

## Introduction

Preeclampsia is a serious condition affecting 2% to 8% of pregnancies and is characterized by gestational hypertension (systolic blood pressure>140 or diastolic blood pressure >90) and proteinuria, or other symptoms of end-organ damage occurring after 20 weeks of gestation [1]. It is caused by narrowed spiral artery formation, leading to placental hypoperfusion and increased antiangiogenic factors that result in maternal endothelial damage [2]. This condition is associated with high maternal morbidity and mortality, with approximately 50,000 deaths occurring annually worldwide [1]. Although preeclampsia can be managed with antihypertensive drugs, delivery of the fetus is the most effective treatment method and accounts for 15% of preterm births [1].

Less than 3% of cases progress to eclampsia, which is diagnosed by the presence of preeclampsia and new-onset seizure activity [3]. The perinatal mortality rate of eclampsia in developed countries ranges from 5.6% to 11.8% and is as high as 14% in developing countries [4]. Most hypertensive disorders in pregnancy resolve within three months postpartum, but many complications can arise during that time. Some complications of preeclampsia/eclampsia (PE/E) include disseminated intravascular coagulopathy (DIC), pulmonary edema, adult respiratory distress syndrome (ARDS), seizures, and cerebral hemorrhaging [4]. Multi-organ dysfunction commonly occurs, involving the eyes, liver, kidneys, and brain. The effect of preeclampsia on the brain may be reversible or permanent and manifests as (i) eclampsia (presence of seizures), (ii) cerebral edema, (iii) stroke, (iv) brain herniation, and (v) cerebral hemorrhage, accounting for 40% of maternal deaths [5].

Posterior reversible encephalopathy syndrome (PRES) is a rare reversible complication associated with PE/E. Due to the uncommon nature of PE/E, the exact prevalence of PRES is not well-known. However, studies with small sample sizes have found varying prevalence rates between 10% and 90% [6]. In a study by Mayama et al., out of 39 pregnant patients with neurological symptoms, 12 of 13 with eclampsia and five of 26 with preeclampsia developed PRES [7]. This condition can occur during the pre-, peri-, or postpartum period and is characterized by vasogenic cerebral edema predominantly in the posterior occipital and parietal lobes, leading to headache, seizures, altered mental status, vision loss, and focal neurological deficits [6]. 

## Case presentation

A 22-year-old female patient was brought to the medical emergency room of a tertiary care hospital after being referred from a local hospital with a history of generalized tonic-clonic seizures, altered state of consciousness, and left-sided weakness for 12 hours. The patient had undergone a cesarean section delivery two days prior. During her third trimester of pregnancy, the patient developed eclampsia with hypertension and episodes of seizure activity. No other significant medical history was elicited.

Upon physical examination, the patient exhibited a blood pressure of 170/100 mmHg, a pulse rate of 88 beats per minute, a respiratory rate of 18 breaths per minute, and an oxygen saturation level of 96% on room air. The patient was afebrile. Neurologically, the patient didn’t respond to commands but did exhibit localization to painful stimuli. Assessment of the plantar reflex was remarkable for a pathological Babinski sign on the left side and deep tendon reflexes were present but exhibited sluggishness.

Notably, there were no signs of meningeal irritation, as evidenced by the absence of neck stiffness or Kernig’s or Brudzinski’s signs. However, the power in both limbs could not be ascertained due to the patient’s altered mental state. The remainder of the systemic examination did not yield any notable neurological findings. Laboratory reports revealed normal hemoglobin 11.8g/dL (Normal: 11.0-15.5 g/dL), total leukocyte count 10.6 x 10^3^/μL (Normal: 4.0-11.0 x 10^3^/uL), platelet counts 153 x 10^3^/μL (Normal: 150-400 x 10^3^/uL), INR 1.0 (<1.1). Urinalysis was remarkable for the presence of a urinary tract infection (UTI) characterized by pyuria with 8-10 pus cells/high-power field (Normal: 0-5 cells/high-power field), mild hematuria with red blood cells 10-12/high-power field (Normal: 0-5 cells/high-power field), proteinuria +2 (Normal: nil), and urobilinogen +2 (Normal: nil). Erythrocte Sedimentation Rate (ESR), renal function tests, and liver function tests were within the normal range. Other blood tests, autoantibodies (ANA, ANCA, anti-dsDNA), viral markers (for Hepatitis B and Hepatitis C), neoplastic markers, cerebrospinal fluid analysis, chest radiographs, and arterial blood gas analysis were normal. Ultrasonography (USG) of the kidney, ureter, bladder, and pelvis revealed an unremarkable scan.

**Figure 1:**
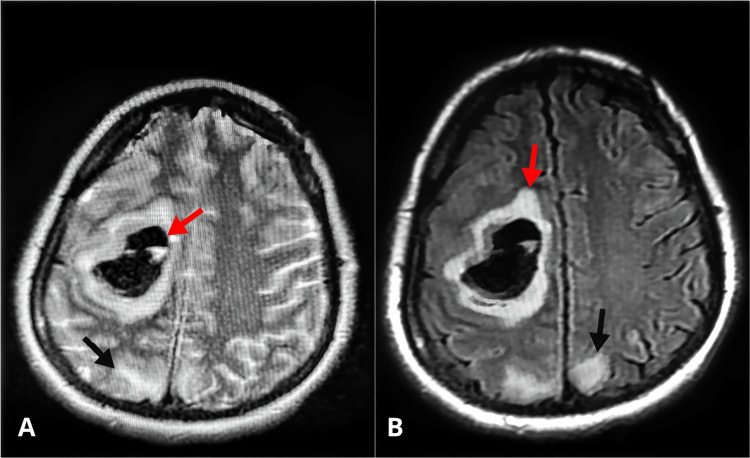
Axial T2 FSE view (A) and FLAIR view (B) Image 16/21: Red arrows indicate intracerebral hemorrhage while black arrows are suggestive of PRES FSE: Fast spin echo; FLAIR: Fluid-attenuated inversion recovery; PRES: Posterior reversible encephalopathy syndrome

MRI of the brain with contrast showed a well-defined altered signal intensity area appreciated in the right fronto-parietal lobe with surrounding edema measuring 3.3 x 2.6 cm (anteroposterior * transverse). The returning signals were isointense on T1W1 and hypointense on T2W1 & T2 fluid-attenuated inversion recovery (FLAIR) and showed no restriction on Diffuse Weighted Imaging/Apparent Diffusion Coefficient images and no post-contrast enhancement. Features most likely intracerebral bleed (acute stage). Multiple subcortical T2W1 and T2 FLAIR hyperintensities were appreciated in bilateral occipital lobes, suggesting PRES (Figures 1A, 1B).

Magnetic resonance venography was unremarkable with normal sinuses and superficial veins. The patient was admitted to the high dependency unit and managed with intravenous fluids, i.e., normal saline and mannitol 10% w/v. The patient was diagnosed as having acute intracerebral bleed so Mannitol was given in order to both improve cerebral blood flow (CBF) and decrease intracranial pressure in both the hemorrhagic and non-hemorrhagic hemispheres. Anti-hypertensive (labetalol) was given to manage the patient’s elevated blood pressure (170/100 mmHg). The patient had presented with a history of generalized tonic-clonic seizures so anti-epileptics (sodium valproate and levetiracetam) were given in order to prevent further episodes of fits. In addition, antibiotics were also added to the treatment regimen to treat her associated condition of UTI. During her stay in the hospital, her blood pressure was constantly monitored. The patient improved symptomatically with a return to normal sensorium and vital signs. The patient was discharged upon request with antihypertensives after 12 days of admission. Follow-up after 10 days was uneventful.

## Discussion

PRES is associated with PE/E. However, other related conditions include: (i) organ transplantation, especially with the use of immunosuppressants cyclosporine or tacrolimus, (ii) autoimmune diseases such as systemic lupus erythematosus (SLE) and granulomatosis with polyangiitis, (iii) chemotherapy use, (iv) infections, and (v) renal disorders such as glomerulonephritis [8]. In this case, the patient's history and labs were remarkable for eclampsia extending into the postpartum period and a UTI. The exact mechanism behind the development of PRES in the setting of PE/E is unknown and controversial; however, studies have theorized a couple of possibilities. The brain requires constant CBF, which can be regulated by changes in cerebral vascular resistance (CVR) [5]. One hypothesis is that cerebral vasoconstriction and vasospasm in PE/E increase the CVR, leading to hypoperfusion and ischemic damage of the brain parenchyma, resulting in cytotoxic edema [9]. Another theory suggests that in preeclampsia, the mean arterial pressure (MAP) exceeds the CBF autoregulatory range when systolic blood pressure reaches 170-190 mmHg [5]. However, in some cases, this may occur at lower systolic blood pressures. This increases intravascular pressure, overcomes arterial vasoconstriction, thus decreasing the CVR, resulting in hyperperfusion and subsequent endothelial damage, blood-brain barrier (BBB) disruption, and cerebral vasogenic edema [5,9]. A study by Williams et al. found that CBF velocity increased within one week postpartum in women with preeclampsia [10]. Another study by Belfort et al. suggested that in severe cases of preeclampsia, the cerebral perfusion pressure is remarkably higher than in mild preeclampsia or normotensive pregnancies [11].

PRES due to PE/E can arise at variable times after the twentieth week of pregnancy and extend into the postpartum period [12]. Onset may be acute, with symptoms appearing within a few hours or days, or subacute, with symptomatic presentation evolving over a period of weeks [12]. The effect of edema formation in the brain leads to a diverse set of symptoms with varying severity depending on which area is affected [6]. PRES predominantly affects the posterior portion of the brain more than the anterior portion. Studies suggest that this may be because there is a higher density of sympathetic innervation by the superior cervical ganglion in the anterior circulation than in the posterior circulation, making it more resistant to changes in pressure [12]. Edema involving the posterior parietal and occipital lobes leads to visual symptoms such as impaired visual acuity, visual field deficits such as homonymous hemianopsia, and cortical blindness [9]. Additionally, other symptoms include (i) altered mental status, which may range from mild confusion to agitation and coma, (ii) seizures, (iii) headache, (iv) nausea and vomiting, and (v) focal neurological deficits [9]. Severe cases may lead to seizure activity, status epilepticus, and coma, and although PRES is reversible, if not managed immediately, it can lead to irreversible parenchymal injury and physical or mental disabilities [6]. The risk of developing severe PRES is increased in postpartum eclampsia, recurrent seizure activity, cesarean delivery, and labetalol use [13]. In this case, the patient had a history of PE/E treated with labetalol, recurrent seizure activity, and a cesarean delivery, which increased her risk of PRES with severe features. There were no visual manifestations in this case; however, the patient exhibited generalized tonic-clonic seizure activity, decreased consciousness, and abnormal neurological findings, including a positive Babinski reflex of the left foot with mildly hypoactive deep tendon reflexes. Untreated PRES can lead to life-threatening complications like cerebral hemorrhage and herniation and refractory status epilepticus [14].

Due to the high morbidity risk of PRES and its complications, a high index of clinical suspicion is required. A thorough patient history and serological tests can rule in or out autoimmune disorders, medication use, or infectious etiologies, and imaging with MRI can aid in confirming the diagnosis. MRI is the gold standard diagnostic test for PRES since CT imaging may be normal, except in the case of concomitant cerebral hemorrhage [15]. The MRI findings of PRES may vary; however, the most commonly described finding in the literature is bilateral cortical and subcortical hyperintensities in vascular watershed areas in the posterior regions of the brain on T2 and FLAIR-weighted MRI [15,16]. T1-weighted MRI and CT scan may show hypointense signals and decreased attenuation in the affected area, respectively [16]. Studies suggest that the cerebral edema found on MRI is more consistent with vasogenic edema than cytotoxic edema, which supports the autoregulation theory [5]. Although parieto-occipital involvement is more common, PRES has been found to affect other areas of the brain, such as the frontal and temporal lobes, cerebellum, and brainstem [6]. A study by Raman et al. analyzed the location distribution of lesions on MRI in 92 patients with PRES [16]. They found that 100% of the lesions were in the parieto-occipital lobes, 30.4% were located in the frontal lobes, 22% in the basal ganglia, 17.39% in the cerebellum, 9% in the brainstem, 8.69% in the temporal lobe, and 4% in the thalamus [16]. 43% of the cases showed restricted diffusion, and 9% had hemorrhaging [16]. Hefzy et al. discovered three types of hemorrhage in 151 patients with PRES due to various etiologies [17]. They included: (i) minute hemorrhages (<5mm in size), (ii) subarachnoid blood in the cortical sulci, and (iii) parenchymal hematoma. They also discovered that cerebral hemorrhage was most frequent in patients with a history of transplantation with immunosuppressant use (22%) and least frequent in patients with eclampsia (5.6%) [17]. The patient in that case with eclampsia-associated PRES and cerebral hemorrhage had severe hypertension with a MAP of more than 116 mmHg [17]. In this case, our patient had subcortical hyperintensities in the bilateral occipital lobe accompanied by a parenchymal hematoma in the right fronto-parietal lobe. Her MAP was 123 upon blood pressure assessment, which is similar to the findings of the aforementioned study.

Delayed treatment of PRES and its complications increases the risk of irreversible neurological deficits, which occur in 15% of patients, and death in 3% to 6% of cases [9,18]. The main goal of treatment is to remedy the underlying issue, which should lead to PRES resolution. In PE/E-associated PRES, magnesium therapy during pregnancy, anticonvulsants, and blood pressure control with antihypertensives are required [6]. Studies suggest that the blood pressure should be reduced by at least 25% with continuous monitoring and management of fluctuations [18,19]. Nitroglycerin should be used with caution in some cases since it may worsen PRES symptoms in patients with PE/E [19]. The patient in this case was managed adequately with IV fluids, antihypertensives, anticonvulsants, and due to the presence of a UTI, antibiotics were added to the regimen. Early treatment normally results in complete resolution within a week for most cases, with a 5% to 10% chance of recurrence [18]. Strict blood pressure management and close follow-ups are required to mitigate this possibility. In this case, there was complete resolution of the patient's symptoms. On her follow up visit 10 days after discharge, vital signs were normal, and no signs of recurrence were noted.

## Conclusions

PE/E is a life-threatening condition that can be detrimental to both the mother and fetus. One major complication that may arise in these patients is PRES which remains poorly understood and manifests with such a wide range of features that it may be diagnosed when symptoms are severe, and permanent neurological deficits are imminent. In PE/E, the presence of the characteristic occipital cortical or subcortical hyperintensities on MRI confirms the diagnosis. PE/E-associated PRES can arise at any time, and may extend into the postpartum period emphasizing the need for close peri- and postpartum follow up, especially in cases where blood pressure continues to be elevated. Physicians should be aware of this condition and its diverse range of symptoms, especially in patients with multiple risk factors. This will allow the facilitation of a timely diagnosis with MRI and management to treat the underlying etiology to enhance the patient's quality of life.

## References

[1] Roberts JM, Gammill HS (2005). Preeclampsia: recent insights. Hypertension.

[2] Amaral LM, Wallace K, Owens M, LaMarca B (2017). Pathophysiology and current clinical management of preeclampsia. Curr Hypertens Rep.

[3] (2023). Eclampsia. https://my.clevelandclinic.org/health/diseases/24333-eclampsia..

[4] Sharara HA, Shaikh N, Ummunnisa F, Aboobacker N, Tamimi HA (2023). Changes in trends and outcomes of eclampsia: a success story from Qatar. Qatar Med J.

[5] Hammer ES, Cipolla MJ (2015). Cerebrovascular dysfunction in preeclamptic pregnancies. Curr Hypertens Rep.

[6] McDermott M, Miller EC, Rundek T, Hurn PD, Bushnell CD (2018). Preeclampsia: association with posterior reversible encephalopathy syndrome and stroke. Stroke.

[7] Mayama M, Uno K, Tano S (2016). Incidence of posterior reversible encephalopathy syndrome in eclamptic and patients with preeclampsia with neurologic symptoms. Am J Obstet Gynecol.

[8] Bartynski WS, Boardman JF (2007). Distinct imaging patterns and lesion distribution in posterior reversible encephalopathy syndrome. Am J Neuroradiol.

[9] Sudulagunta SR, Sodalagunta MB, Kumbhat M, Settikere Nataraju A (2017). Posterior reversible encephalopathy syndrome (PRES). Oxf Med Case Reports.

[10] Williams KP, Galerneau F, Wilson S (1998). Changes in cerebral perfusion pressure in puerperal women with preeclampsia. Obstet Gynecol.

[11] Belfort MA, Varner MW, Dizon-Townson DS, Grunewald C, Nisell H (2002). Cerebral perfusion pressure, and not cerebral blood flow, may be the critical determinant of intracranial injury in preeclampsia: a new hypothesis. Am J Obstet Gynecol.

[12] Fischer M, Schmutzhard E (2017). Posterior reversible encephalopathy syndrome. J Neurol.

[13] Shaikh N, Nawaz S, Ummunisa F (2021). Eclampsia and posterior reversible encephalopathy syndrome (PRES): A retrospective review of risk factors and outcomes. Qatar Med J.

[14] Munirah MP, Mohamad N, Norhayati MN, Nurul Azman A (2023). Posterior reversible encephalopathy syndrome: a conundrum of nephrotic syndrome complication. Electronic J Gen Med.

[15] Hobson EV, Craven I, Blank SC (2012). Posterior reversible encephalopathy syndrome: a truly treatable neurologic illness. Perit Dial Int.

[16] Raman R, Devaramane R, Jagadish GM, Chowdaiah S (2017). Various imaging manifestations of posterior reversible encephalopathy syndrome (PRES) on magnetic resonance imaging (MRI). Pol J Radiol.

[17] Hefzy HM, Bartynski WS, Boardman JF, Lacomis D (2009). Hemorrhage in posterior reversible encephalopathy syndrome: imaging and clinical features. Am J Neuroradiol.

[18] (2023). Posterior Reversible (Leuko) Encephalopathy Syndrome (PRES) - Increasingly Linked to Medicines. https://www.medsafe.govt.nz/profs/PUArticles/March2017/PERSLinkedtoMedicines.htm.

[19] Achar SK, Shetty N, Joseph TT (2011). Posterior reversible encephalopathy syndrome at term pregnancy. Indian J Anaesth.

